# Biosimilar uptake by British local formularies: a cross sectional study

**DOI:** 10.1007/s11096-017-0523-6

**Published:** 2017-09-04

**Authors:** Saja Alnahar, Rachel A. Elliott, Murray D. Smith

**Affiliations:** 1 Division of Pharmacy Practice and Policy, School of Pharmacy, University of Nottingham, East Drive, University Park, Nottingham, NG7 2RD UK; 2 Manchester Centre for Health Economics, Division of Population Health, Health Services Research and Primary Care, School of Health Sciences, Faculty of Biology, Medicine and Health, The University of Manchester, Oxford Road, Manchester, M13 9PL UK; 3 Community and Health Research Unit, School of Health and Social Care, University of Lincoln, Lincoln, UK

**Keywords:** Biosimilars, Biological medicines, Great Britain, Local formularies

## Abstract

*Background* Biological medicines are starting to lose their patent protection, so similar, inexact copies (biosimilars) are being developed and licensed. The high acquisition costs of biologics for healthcare providers could be reduced by switching to biosimilars, thus alleviating budgetary pressures and increasing patient access. Therefore, the acceptance of biosimilars by prescribers in Great Britain (GB; England, Scotland, Wales) needs to be described and understood. *Objective* To determine uptake of the first wave of biosimilars (somatropin, epoetin, filgrastim) by local formularies (lists of preferred medicines for prescribing in local healthcare settings). *Settings* This study targeted local formularies in GB. *Method* In November 2014, local formularies (medicines formularies of Acute Trusts and Health Boards in GB) were screened for their approach to listing of biologics and their biosimilars as well as recommendations on usage of these pharmaceuticals. *Main Outcomes Measures* Listing frequencies of biosimilars. *Results* One hundred and forty-six British local formularies were screened. Amongst the 80% of formularies in which brand names were specified, biosimilar filgrastim was the most frequently listed when compared to the other targeted biosimilars. Biosimilars were listed in preference to reference biologic medicine in 49% of local formularies for filgrastim, 11% for somatropin and in only 6% for epoetin. *Conclusion* Although the market for biosimilars can act in parallel to the generic market, their uptake measured using local British formularies was less than what is expected given that the British market for medicines has a strong focus on generics. Finally, geographical variability within GB requires further investigation.

## Impacts on practice

- As the use of brand names in prescribing and reporting adverse drug reactions is part of active monitoring of biological medicines safety, including the biosimilar medicines, local formularies must actively list these medicines using the brand name;
- Specifying preferred brand for prescribing facilitates and promotes local formularies in guiding cost effective and rational medicines utilisation.

## Introduction

Biological medicines (BMPs) provide an innovative method for treating chronic and life-threatening diseases such as cancers, rheumatoid arthritis and multiple sclerosis. Their rapid adoption into clinical practice has resulted in a substantial share of the pharmaceutical market; for example, in 2012–2013, 27% of total pharmaceutical sales in Europe were BMPs [1]. Moreover, eight of the top ten bestselling pharmaceuticals in 2013 in Europe were BMPs. It is expected that BMPs will maintain the same market share into 2016 [2, 3] and likely beyond. BMPs have significantly improved health and disease outcomes, but their widespread utilisation is financially challenging to healthcare providers worldwide [4].

In 2013–2014, in England, seven out of the top ten most prescribed medicines in terms of cost to the National Health Service (NHS) were BMPs [5]. Notable though is that all seven will lose their patent protection by 2018 [6]. Patent expiry creates an opportunity for developing and licensing similar copies of off-patent BMPs-called biosimilar medicines (BSPs) [7]. Unlike conventional generic medicines, BSPs are not identical but similar copies of their reference BMP (R-BMP). The inability to produce identical copies is related to the complex and heterogenic molecular structure of these medicines, and being produced by not fully controllable living systems [4, 8].

In their analysis of the European BSP market, Rovira et al. reported that BSPs offer price reductions of between 10 and 35% [9]. Although this is a modest price reduction in comparison to conventional generic medicines, the overall cost saving to health service providers is expected to be significant due to the high unit costs and extensive use of BMPs [9]. The European Generic Medicines Association reported that the European Union might save upwards of €1.6 billion/year for the scenario in which a 20% price reduction occurs on five off-patent BMPs [10]. Furthermore, it has been suggested that the availability of BSPs might alter medical practice and increase compliance to clinical guidelines [11, 12].

In Europe, BSPs can be launched after being centrally assessed and licensed by the European Medicines Agency (EMA) for safety, quality and efficacy [8, 11]. While the EMA mandates full quality submission in order to license a BSP, it does permit evidence based extrapolation of the clinical outcomes from one tested indication to closely related others [13, 14].

In Great Britain (GB), the Medicines and Healthcare products Regulatory Agency (MHRA) directs prescribers to use brand name in prescribing any BMP and its BSPs [15]. Brand name-based prescribing and reporting facilitates capture of safety issues related to BMPs including BSPs [15].

Despite being a generically-driven market [16], the uptake of BSPs in Britain seems to have been relatively limited when compared to other European countries [14]. According to the British Generic Manufacturers Association factors related to physicians’ lack of confidence in prescribing BSPs, absence of encouraging national policies and banned substitution at pharmacy level have resulted in slow adoption of BSPs [14]. Lack of confidence might relate to physicians questioning the safety and efficacy of BSPs [11]. This might be at least partly explained by the abridged approach the EMA follows in assessing clinical efficacy of BSPs [13]. One exception is granulocyte-colony stimulating factor (G-CSF)-filgrastim BSP, which is achieving an uptake rate similar to that of conventional generic medicinal products [2].

One approach to evaluating prescribers’ acceptance of BSPs is through assessing uptake of BSPs in local medicines formularies. The National Institute for Health and Care Excellence (NICE) defines local formularies as “the output processes to support the managed introduction, utilisation or withdrawal of healthcare treatments within a health economy, service or organisation” [17]. Local formularies are working documents that are subject to regular review and change. They tend to be robust on treatments used frequently and weak on occasionally used ones (that need to be available for a complete and comprehensive service). Local formularies can act as prescribing guiding tool at local settings [18]. Heal et al. (2004) reported that more than 80% of their study participants were guided by local formularies [19]. Accordingly, assessing BSP listing in local formularies might reflect acceptance of these products by prescribers.

## Aim of the study

To determine uptake acceptance of first wave BSPs licensed and marketed in GB prior to 2015 [somatropin (HGH), epoetin (EPO) and filgrastim (G-CSF)] by local formularies.

## Ethics approval

Ethical approval was not needed as local formularies throughout the UK are publically available documents included in the freedom of information scheme.

## Method

During November–December 2014, a list of 157 Acute Trusts (accountable organisations within NHS England that manage and control the performance, services quality and financial efficiency of clusters of hospitals in England) was acquired from the NHS England website two Acute Trusts were excluded as one was dissolved and the other one was still in process of developing a formulary list. Details of the 14 Regional Health Boards in Scotland and 7 Local Health Boards in Wales (accountable organisations within NHS Scotland and NHS Wales that are responsible for the delivery of healthcare services to the local population) were acquired from Scottish Government and NHS Wales websites. Northern Ireland was excluded as there was an initiative underway to develop a joint formulary for the whole country, and some of its chapters were still under development.

The most recent versions of local medicines formularies were downloaded and examined for their listing of targeted R-BMPs and BSPs according to the criteria given in Table 1. These criteria enabled assessment of: (1) uptake of BSPs, (2) compliance to the MHRA recommendation on brand name prescribing of BMPs, and (3) the consideration given to population or indication-specific recommendations.

**Table 1:** **-** Formulary assessment criteria

No.	Assessment criterion	Definition	
1.	Is this formulary a joint formulary? (yes/no)	Formulary list that is developed and/or used by more than one healthcare setting (primary and/or secondary care)	
2.	Is the targeted medicine listed? (yes/no)	BNF chapter 6.5.1*: somatropin (HGH)	
		BNF chapter 9.1.3**: epoetin alfa (EPO)	
		BNF chapter 9.1.6***: filgrastim (G-CSF)	
3.	How it is listed? (formulary/non-formulary)	Formulary	Medicinal product that is routinely available for prescription
		Non-formulary	Medicinal product that is not routinely available for prescription. However, if it is deemed needed, it will be made available for the patient
4.	What is the listing approach? (brand name/INN/both)	As per brands listed in BNF chapters 6.5.1, 9.1.3 and 9.1.6	
5.	What are the listed brands? (R-BMP/BSP/both)	BNF chapter 6.5.1: 1 R-BMP and 1 BSP	
		BNF chapter 9.1.3: 1 R-BMP and 2 BSPs	
		BNF chapter 9.1.6: 1 R-BMP and 3 BSPs	
6.	What is the preferred type? (R-BMP/BSP/not specified)	Being the only listed brand/type; Clearly stated that this brand/type is the preferred for prescription; or Brand/type listed as formulary while other brands/type listed as non-formulary.	
7.	Are there clear restrictions in prescribing? (yes/no)	Whether or not there are specified restrictions in terms of who can prescribe these products and/or prescribing settings i.e. primary or secondary care	
8.	Is there specified brand of choice? (yes/no)	If it is clearly stated that a specific brand is considered as the brand of choice	
9.	Are there special population considerations? (yes/no)	If there are considerations related to patients’ age, medical history and/or life style factors that might affect type/brand of preference (R-BMP or BSP)	
10.	Are there special indications’ considerations? (yes/no)	If there are considerations related to indications that might affect type/brand of preference (R-BMP or BSP)	

*BNF* British National Formulary 68th Edition; *BMP* biological medicinal product; *BSP* biosimilar medicinal product; *INN* international non-proprietary name (molecular name); *R-BMP* reference biological medicinal product

* 6.5.1: drugs used in hypothalamic and anterior pituitary hormones and anti-oestrogen; 1 R-BMP and 1 BSP brand

** 9.1.3: drugs used in hypoplastic, haemolytic, and renal anaemias; 1 R-BMP and 2 BSP brands

*** 9.1.6: drugs used in neutropenia; 1 R-BMP and 3 BSP brands

The assessment process started by examining whether the listing approach of R-BMP and BSPs was based on using brand name, molecular name or both, as per the British National Formulary (BNF) 68th edition. Then the availability of prescribing guidance in terms of speciality of the prescriber and/or the clinical settings was recorded. Finally, formulary entries were classified and compared according to preferred brand for prescribing. Formulary uptake of targeted BMPs and BSPs was analysed using descriptive statistics in an Excel^®^ 2010 spreadsheet.

## Results

One hundred and forty-six formularies were identified: 129 in England, 10 in Scotland and 7 in Wales. Forty-three percent (63/146) of these were joint formularies (the term given to a local formulary developed and/or used simultaneously by more than one healthcare providing organisation).

Formulary uptake and listing approaches varied across the three targeted groups. HGH was listed in 126 formularies achieving the highest percentage of medicine listing, EPO achieved the highest brand name based listing with 60 formularies using brand names. Despite BSP G-CSF being the last of the three groups to be authorised by the EMA, it was most commonly listed in preference to the R-BMP. Table 2 provides more details.

**Table 2:** - Results Summary

Investigated attributes	Medicine group		
	Filgrastim (G-CSF)	Epoetin alfa (EPO)	Somatropin (HGH)
Medicine being listed			
Not listed	27 (18%)	31 (21%)	20 (14%)
Listed as formulary	115 (79%)	107 (73%)	120 (82%)
Listed as non-formulary	4 (3%)	8 (6%)	6 (4%)
Total	146	146	146
Clear prescription restrictions			
Yes	78 (66%)	77 (67%)	84 (67%)
No	41 (34%)	38 (33%)	42 (33%)
Total	119	115	126
Listing approach			
INN name	58 (49%)	44 (38%)	64 (51%)
Brand names	49 (41%)	60 (52%)	59 (47%)
Mixed listing	12 (10%)	11 (10%)	3 (2%)
Total	119	115	126
Listed brands			
Both	36 (59%)	21 (30%)	25 (40%)
R-BMP only	11 (18%)	48 (68%)	4 (7%)
BSPs only	14 (23%)	2 (3%)	33 (53%)
Total	61	71	62
Preferred type			
R-BMP	10 (16%)	52 (73%)	29 (47%)
BSPs	30 (49%)	4 (6%)	7 (11%)
Unclear	21 (34%)	15 (21%)	26 (42%)
Total	61	71	62
Specified brand of choice			
Yes	13 (21%)	4 (6%)	9 (15%)
No	48 (79%)	67 (94%)	53 (85%)
Total	61	71	62
Special population considerations^a^			
Yes	3 (5%)	8 (11%)	10 (16%)
No	58 (95%)	63 (89%)	52 (84%)
Total	61	71	62
Special indication considerations			
Yes	9 (15%)	7 (10%)	0 (0%)
No	52 (85%)	64 (90%)	62 (100%)
Total	61	71	62

*BSP* biosimilar medicinal product; *INN* international non-proprietary name (molecular name); *R-BMP* reference biological medicinal product

^a^Factors related to patient population or indication that might affect prescriber brand/product of choice

Variations between the countries of GB were seen, with 27% of English formularies listing BSP G-CSF in preference to the R-BMP, versus 12.5% and 14.3% amongst Scottish and Welsh formularies, respectively. In the cases of HGH and EPO, 7% and 4% of English formularies preferred BSP over R-BMP; respectively, but no Scottish or Welsh formularies did so.

Only six formularies listed at least one BSP from all three targeted groups. There were no instances of BSPs being preferred to R-BMPs across all three targeted groups, but at the opposite extreme there were only two formularies in which R-BMPs were always preferred over BSPs.

## Discussion

In this study, formulary uptake of BSPs in three different groups was employed as an indicator of clinical acceptance of BSPs in GB. The uptake of BSPs by British local formularies was less than what might be predicted from a classical generically driven market for medicines, and there appeared to be geographic variability in uptake which requires further investigation.

Most frequently listed was HGH, the first licensed BSP [20], however the maximum preference for BSPs over R-BMP was achieved by G-CSF BSPs. On the other hand, brand name-based listing was more frequent in the case of EPO. Despite it not being possible to gain a complete understanding of the variations between targeted groups, it might be explained in large part by the molecular nature, therapy duration and/or patients’ demographics, preferences and life style.

Having several other medicinal products within the same group where each has different dosing frequencies, administration devices and storage conditions might affect acceptance of BSPs. For example, one HGH product is a needleless device making it more attractive to paediatric and needle-phobic patients. Another HGH product can be stored out of the refrigerator [21] which might make it the brand of choice for frequent travellers.

G-CSF’s molecular structure and short duration of therapy might increase the acceptance of its BSPs. It is a small, easily characterised non-glycosylated molecule that is indicated for short therapeutic periods [22, 23]. However, special population considerations in terms of patient age and therapeutic indications were flagged in those instances when the R-BMP was listed in preference to BSPs. Specifically, the R-BMP was listed as the preferred product for paediatric patients and for stem cell mobilisation. Reluctance to use BSPs in these settings may be because of the lack of clinical trial evidence of their efficacy and/or safety.

EPO achieved the highest percentage of brand name-based listing. This is most likely due to EPO being a glycosylated protein, in which case each brand has a different glycosylation pattern and hence a different immunogenic profile [24]. In addition, previously reported cases of the life threatening pure red cell aplasia might encourage the use of brand name based listing for EPO whether for the R-BMP or the BSPs [24].

The MHRA has considered several measures in the monitoring of BMPs, including BSPs, such as: demanding brand name-based prescription, and requiring specification of a product’s identifiers (brand name, batch number and manufacturer) in reporting adverse drug reactions. Furthermore, prescribers are directed to inform patients and/or carers about a product’s brand name and batch number [15]. However, more than one third of local formularies did not specify brand names when listing BMPs in the groups examined in this study. Unintentional switching between brands might affect patients’ safety, if not carefully monitored and managed. Such harm might be avoided in the cases of G-CSF and EPO because of their short duration prescription, but possibly not otherwise for HGH which is typically used for prolonged periods.

The limited clinical evidence of BSPs’ efficacy has been highlighted as a factor hindering BSPs’ acceptance in clinical practice [22]. However, the BSP G-CSF brand that was approved based on results of a pharmacokinetics/pharmacodynamics trial in healthy volunteers instead of patients, and in a non-comparative safety-focussed study in patients [25] was recommended for use ahead of R-BMP G-CSF in more local formularies than was any other biosimilar/reference pairing.

As there is a proposal to assign different international non-proprietary name (INN) for BSPs, BSPs’ manufacturers argue that a differing INN might limit the uptake of BSPs by giving suggestion to the view that BSPs are completely different instead of being a highly similar molecule [26]. The effect of different INN was noted in the case of epoetin zeta and epoetin alfa, where despite both being EPO BSP the latter was more often listed than the former. Moreover, 50% of formularies that preferred BSP EPO over the R-BMP have not listed epoetin zeta, while the remaining 50% listed it without brand specification. Of the 27 formularies that did list EPO using its molecular name none included epoetin zeta.

Variations in listing BSPs were observed between England, Scotland and Wales. Although both the Scottish Medicines Consortium and the All Wales Medicines Strategy Group have been actively involved in evaluating BSPs [27, 28], Scottish and Welsh formularies were less likely to list BSPs in preference to the R-BMP compared with their English counterparts.

Research data and analysis indicate that some formularies encourage BSP prescribing. However, variations and lack of specificity in some formularies may suggest a vague understanding of the concept and nature of BMPs in general, and BSPs in particular, by the professionals involved in developing local formularies.

The principal limitation of this study is that the uptake of BSPs has been estimated using local medicines formularies as a proxy for actual prescribing practice. This may not necessarily provide a highly accurate reflection of BSP prescribing, as it is not known to what degree these lists are adhered to.

If equally safe and effective as the originator BMP, lower prices for BSPs could potentially increase access to treatment for more patients, or reduce drug spending in an increasingly constrained financial environment. Engagement is required with prescribers, formulary managers and commissioners to understand the basis for formulary decision-making, identify reasons for variation in prescribing behaviour and develop strategies for more uniform uptake of BSPs. Educational interventions are also needed around adherence to standards of pharmacovigilance to assure drug monitoring and patient safety.

## Conclusion

The results of this study suggest that the uptake of BSPs in Britain is highly variable, and generally less than what is expected, given historically that its market for pharmaceuticals is very much generically driven. Further work is needed to understand why there is such low and variable uptake.
